# Supplementary material for: Cellular microRNA networks regulate host dependency of hepatitis C virus infection
Source: Nat Commun. 2017 Nov 27;8:1789. doi: 10.1038/s41467-017-01954-x (PMC5702611; doi:10.1038/s41467-017-01954-x)
Supplement: Supplementary file 1 — Supplementary Information [file 41467_2017_1954_MOESM1_ESM.pdf]

Supplementary Figures

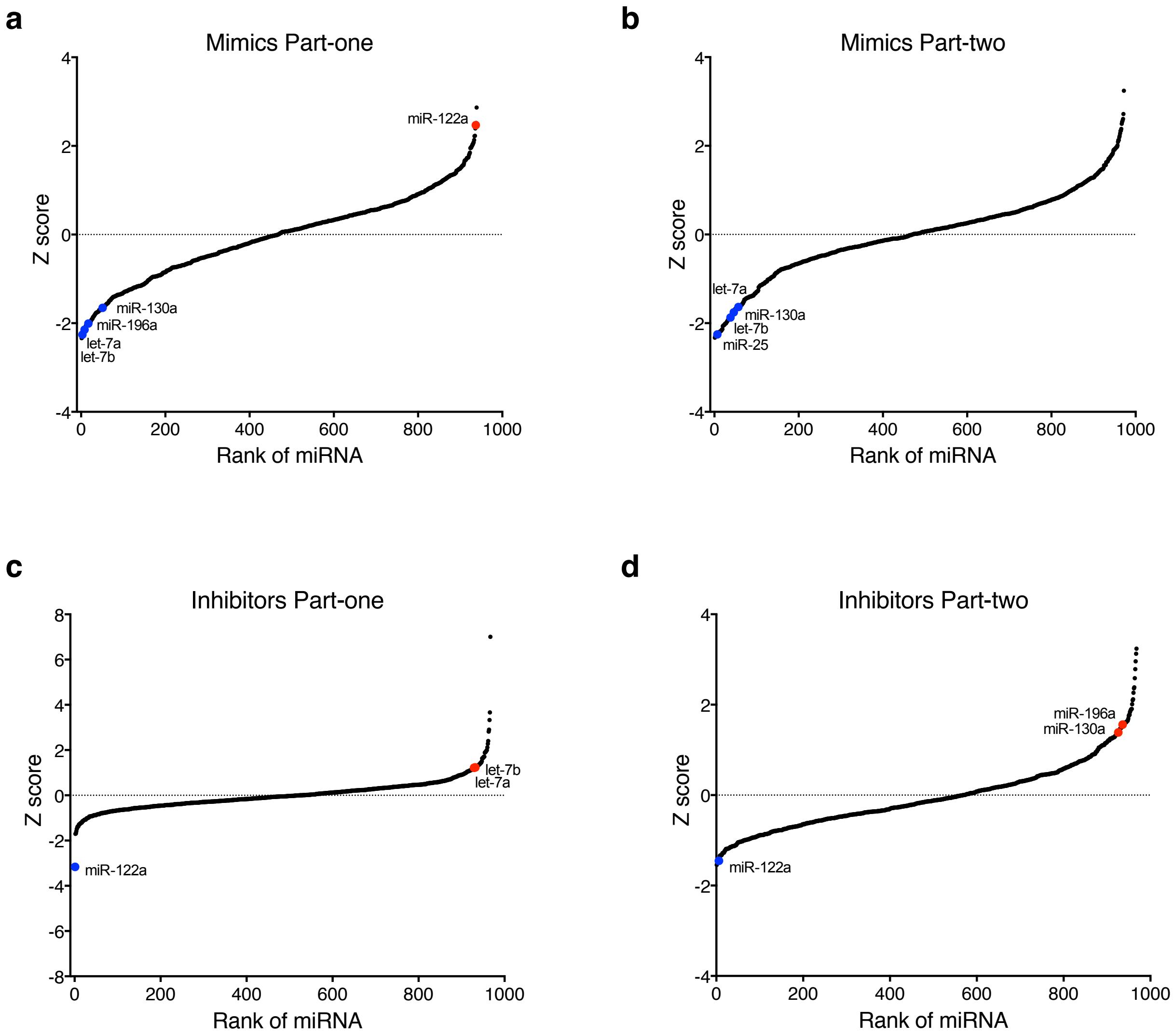

Supplementary Figure 1 Analyses of the miRNA mimic and hairpin inhibitor screens. Graphs depict Z scores of ranked miRNAs from the mimic (**a**,**b**) and inhibitor (**c**,**d**) HCVcc functional screens. Selected miRNA mimics or hairpin inhibitors of interest are shown in blue (antiviral phenotype) or red (proviral phenotype).

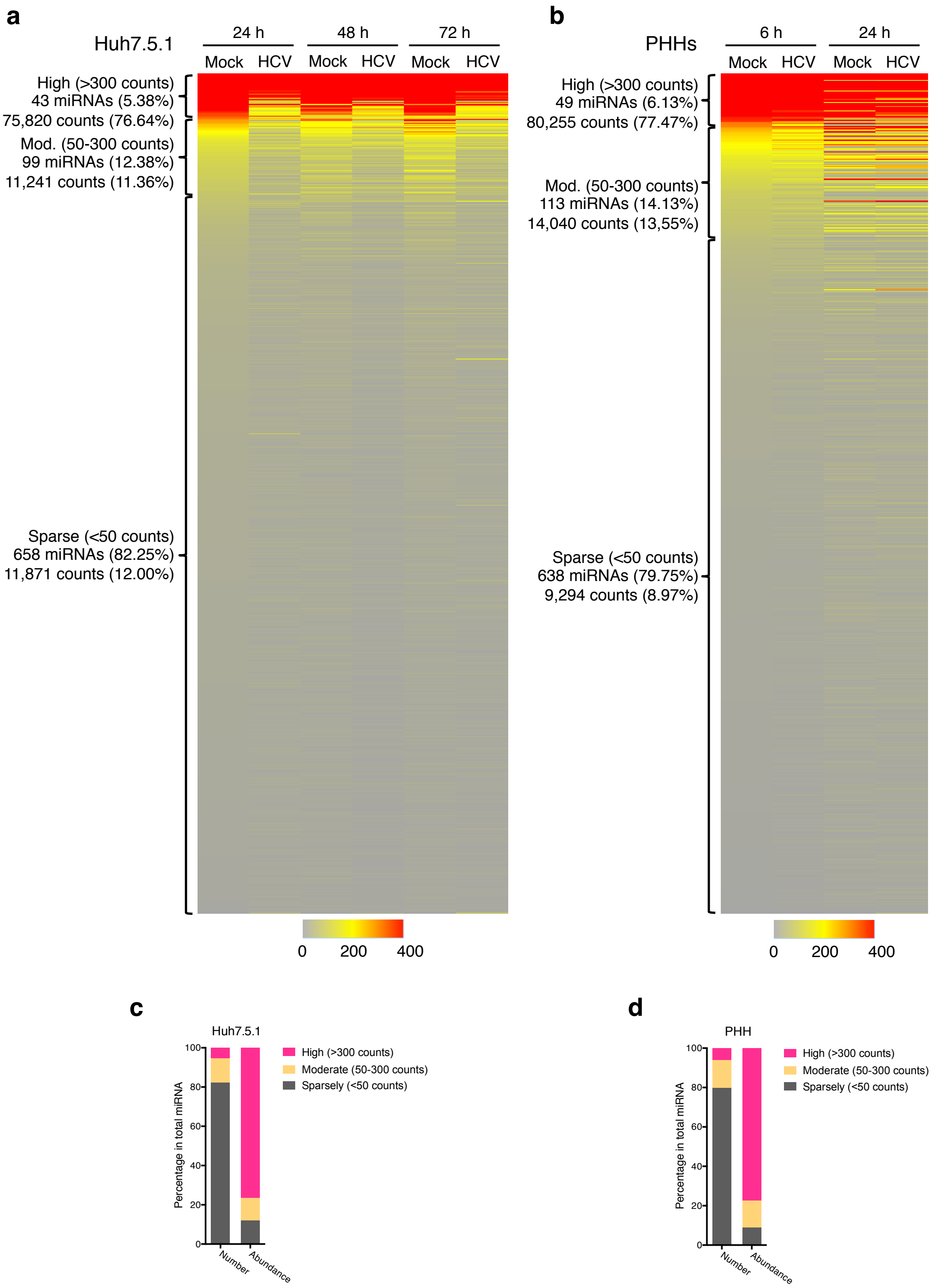

Supplementary Figure 2 NanoString nCounter analyses of the miRNA transcriptome in hepatocytes. (**a**,**b**) Heatmaps illustrating global miRNA expression profiles in mock or HCV-infected Huh7.5.1 cells, 24, 48, and 72 h post-infection (**a**) (see **Supplementary Data 3**) or PHH, 6 and 24 h post-infection (**b**) (see **Supplementary Data 4**). (**c**,**d**) miRNAs are classified as highly, moderately and sparsely expressed groups based on their abundance in Huh7.5.1 cells (**c**) or PHH (**d**). Percentages in total miRNA counts of each group are shown.

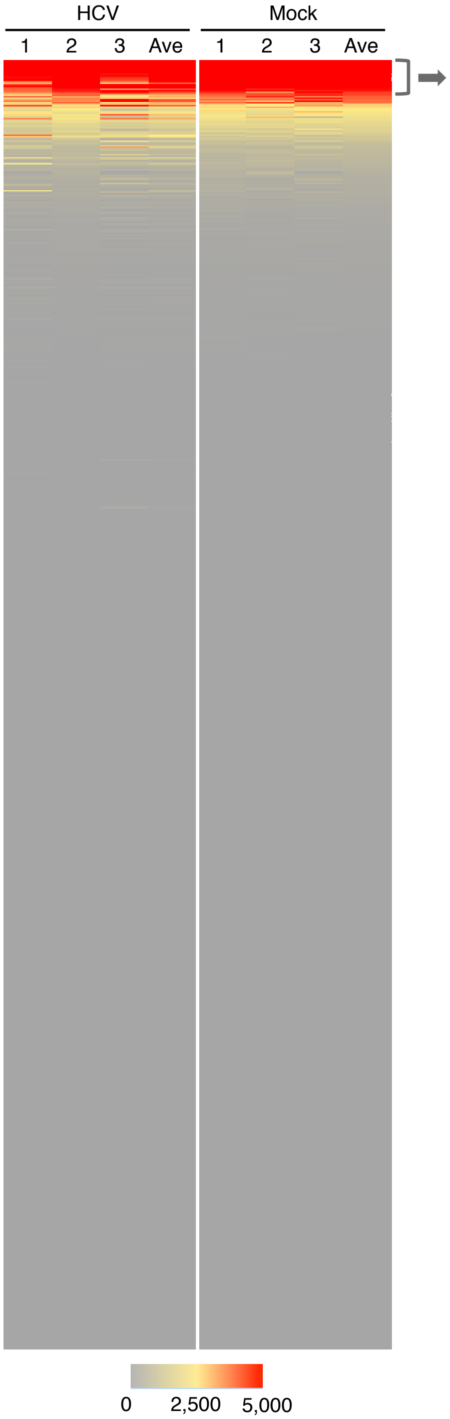

miRNA ID	Expression (Avearge ± SD)	Percentage	Rank
hsa-miR-122	33988 ± 2300	9.69%	1
hsa-miR-1246	26487 ± 1884	7.55%	2
hsa-miR-92a	19169 ± 875	5.46%	3
hsa-miR-21	18397 ± 1656	5.24%	4
hsa-miR-26a	13586 ± 1204	3.87%	5
hsa-miR-25	11599 ± 320	3.31%	6
hsa-miR-20a	11294 ± 486	3.22%	7
hsa-miR-192	10357 ± 758	2.95%	8
hsa-miR-15b	9905 ± 1323	2.82%	9
hsa-miR-17	8990 ± 927	2.56%	10
hsa-miR-92b	8739 ± 1085	2.49%	11
hsa-miR-106a	8299 ± 397	2.37%	12
hsa-miR-16	7919 ± 870	2.26%	13
hsa-miR-23b	6918 ± 276	1.97%	14
hsa-miR-194	6870 ± 746	1.96%	15
hsa-miR-20b	5943 ± 258	1.69%	16
hsa-miR-320c	5646 ± 78	1.61%	17
hsa-miR-638	5397 ± 306	1.54%	18
hsa-miR-320a	5274 ± 176	1.50%	19
hsa-miR-27b	4922 ± 265	1.40%	20
hsa-miR-320b	4849 ± 128	1.38%	21
hsa-miR-423-5p	4330 ± 677	1.23%	22
hsa-miR-191	4299 ± 171	1.23%	23
hsa-miR-148a	4237 ± 308	1.21%	24
hsa-miR-23a	3987 ± 439	1.14%	25
hsa-miR-215	3968 ± 565	1.13%	26
hsa-miR-320d	3868 ± 22	1.10%	27
hsa-miR-26b	3844 ± 1096	1.10%	28
hsa-miR-106b	3810 ± 268	1.09%	29

Supplementary Figure 3 Microarray analysis of the miRNA transcriptome in Huh7.5.1 cells. Cells were either mock infected or infected with HCV for 48 h, before harvested for total miRNA extraction. Heatmap elicits the count of each miRNA determined by the transcriptomics analysis (see **Supplementary Data 5**). The expression levels and percentages of the most abundant miRNAs (>1% of total miRNA counts) are listed in the table on the right panel.

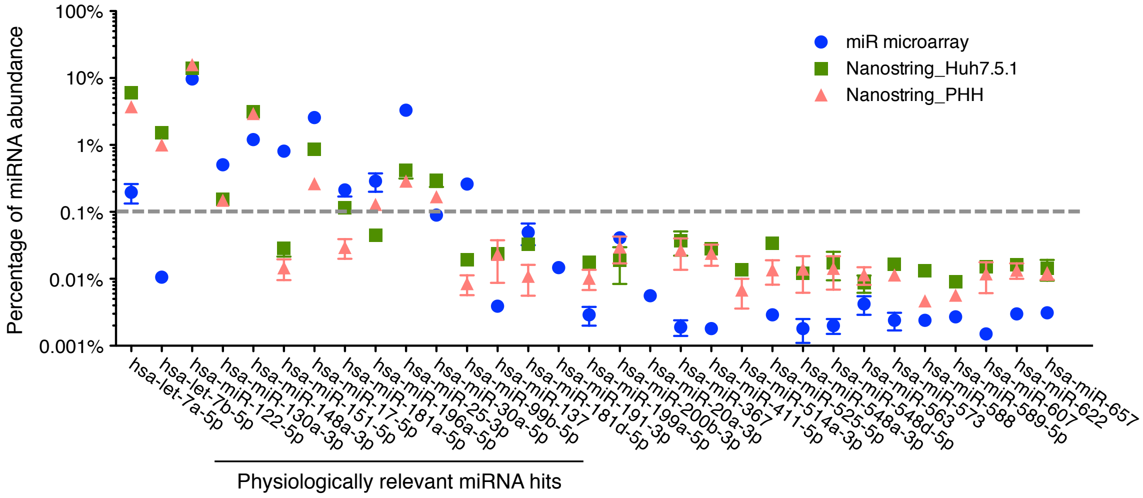

Supplementary Figure 4 Expression profiles of 31 phenotype-specific miRNA hits identified from the mmic and inhibitor screens. miRNA expression levels in Huh7.5.1 cells or PHH measured by the nCounter analysis or microarray were elicited for all 31 phenotype-specific miRNAs. A miRNA is considered physiologically relevant if its expression accounts for higher than 0.1% of the total miRNA counts by at least one method of quantification. Cutoff is depicted as dashed grey line. Error bars represent ± SD of the mean, n=3.

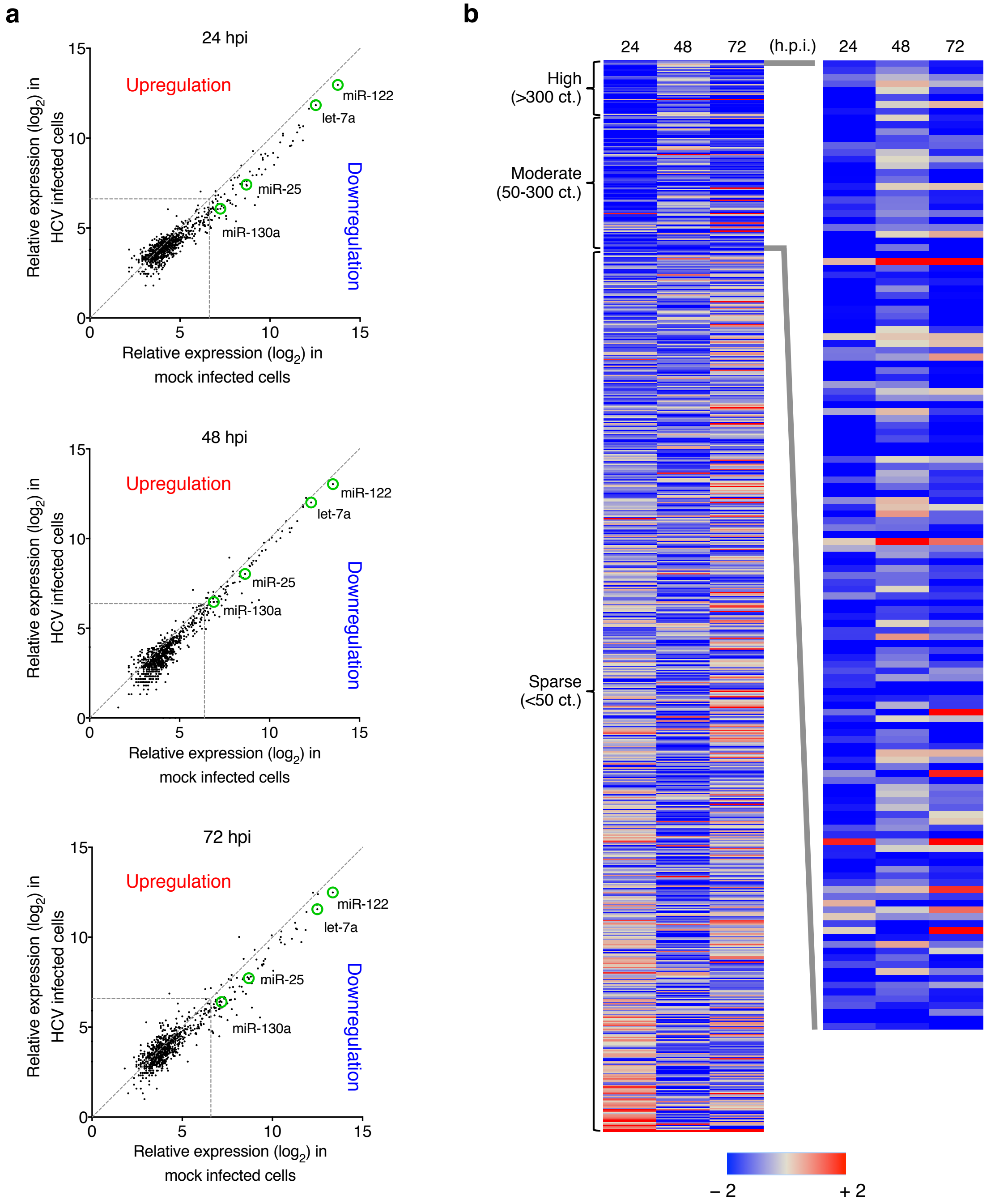

Supplementary Figure 5 HCV infection reduces the overall expression of the miRNA landscape. (**a**) Relative expression of each miRNA in mock- and HCV-infected Huh7.5.1 cells at 24, 48 and 72 h post-infection (hpi), quantified by NanoString nCounter assays. Several downregualted miRNAs of interest are highlighted by green cycles. (**b**) Heatmap illustrating folds of change for all miRNAs (left) and abundantly expressed miRNAs (right) comparing Mock or HCV infection for various indicated time poitns. Fold change in miRNA expression is shown on spectrum from blue (downregulation by HCV) to red (upregulation by HCV).

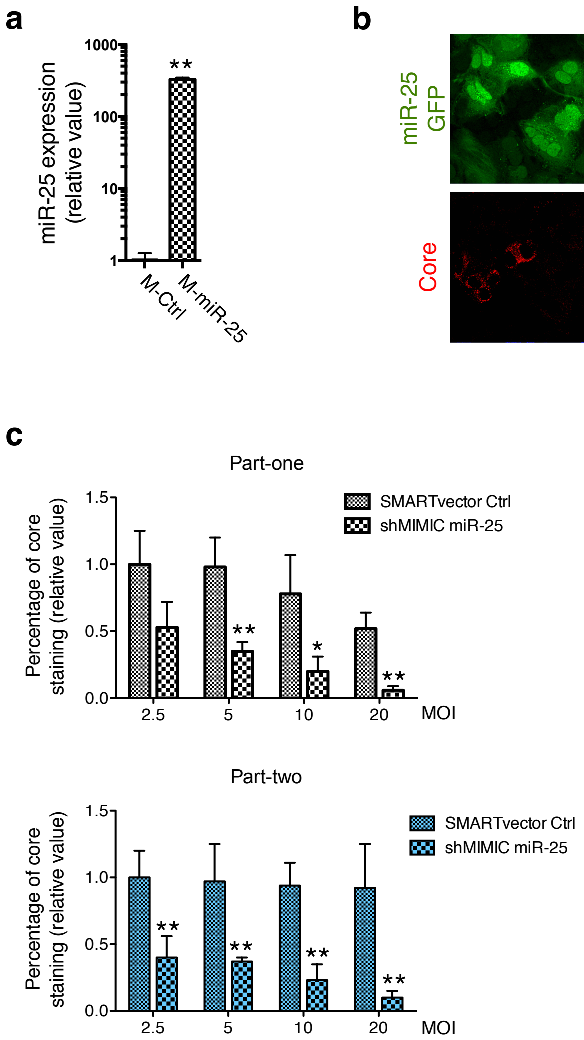

Supplementary Figure 6 miR-25 expression inhibits HCV core production. (**a**) Transfection efficiency of miR-25 mimic overexpression in Huh7.5.1 cells. (**b**) Cells infected with shMIMIC lentiviral miR-25 are resistant to HCV infection, examined by immunofluorescence/confocal microscopy. TurboGFP (in green) serves as a reporter of lentiviral vector-based miR-25 expression. Red, HCV core; blue, nuclei. Scale bars, 100 μm. (**c**) Quantitative analyses of part-one and part-two HCV core immunostaining in Huh7.5.1 cells infected with SMARTvector control or shMIMIC lentiviral miR-25 at various indicated MOI. (**d**) miR-25 expression is unrelated to the extent of fibrosis, assessed by Ishak score, in liver tissues of chronic hepatitis C patients. (**a**,**c**,**d**) Values are normalized relative to the mimic control (**a**), SMARTvector control at the MOI of 2.5 (**c**) or healthy donor liver biopsies (**d**) as 1, and error bars represent SD of the mean, n = 3 (**a**,**c**), or 15 (healthy), 7 (Ishak Score 0-2), 3 (Ishak Score 0-2), or 8 (Ishak Score 0-2) (**d**). *$P < 0.05$, **$P < 0.01$ determined by Student's t test. NS, not significant.

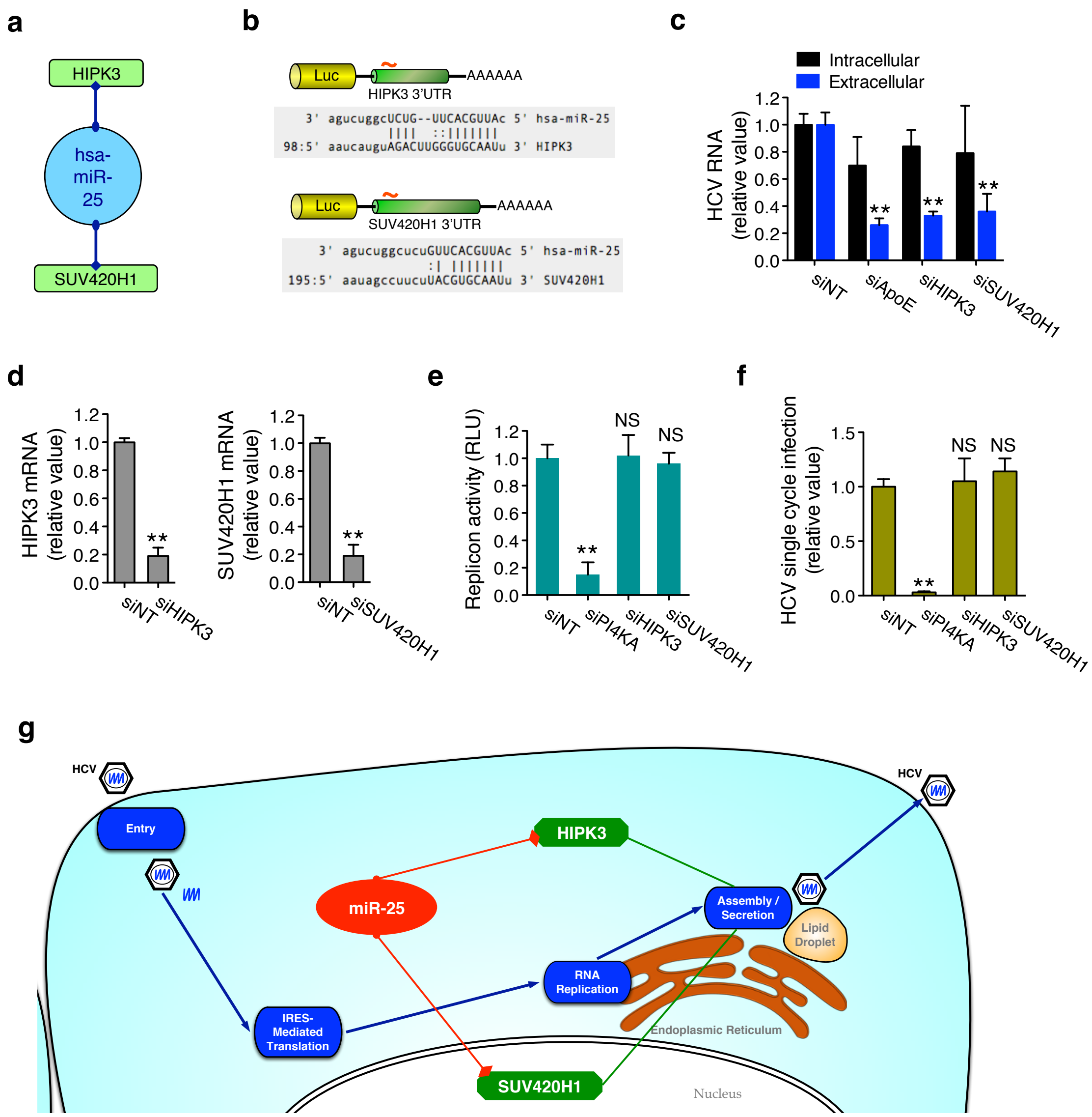

Supplementary Figure 7 miR-25 targets HIPK3 and SUV420H1 to restrict HCV assembly/secretion. (**a**) Predicted targets of miR-25, derived by integrative bioinformatics, functional and transcriptomics analyses, as demonstrated in **Fig. 4f**. (**b**) 3'UTRs of HIPK3 and SUV420H1 possess miR-25 seed matching sites. (**c**) siRNA-mediated silencing of HIPK3 or SUV420H1 in cells inhibits HCV RNA production and secretion. (**d**) Knockdown efficiencies of HIPK3 and SUV420H1 siRNAs. (**e**,**f**) Effects of various indicated siRNAs on HCV replicon activity (**e**) or HCVsc infection (**f**). PI4KA siRNA was used as a positive control for both assays. (**g**) Schematic map illustrating that miR-25 (in red oval) targets HCV host dependency factors HIPK3 and SUV420H1 (in green octagons) to exerts its function in restricting HCV assembly or secretion. Values are normalized relative to the control miRNA or siRNA, and error bars represent SD of the mean, n = 3 (**c**,**d**) or 5 (**e**,**f**). $^{**}P < 0.01$ determined by Student's *t* test. NS, not significant.

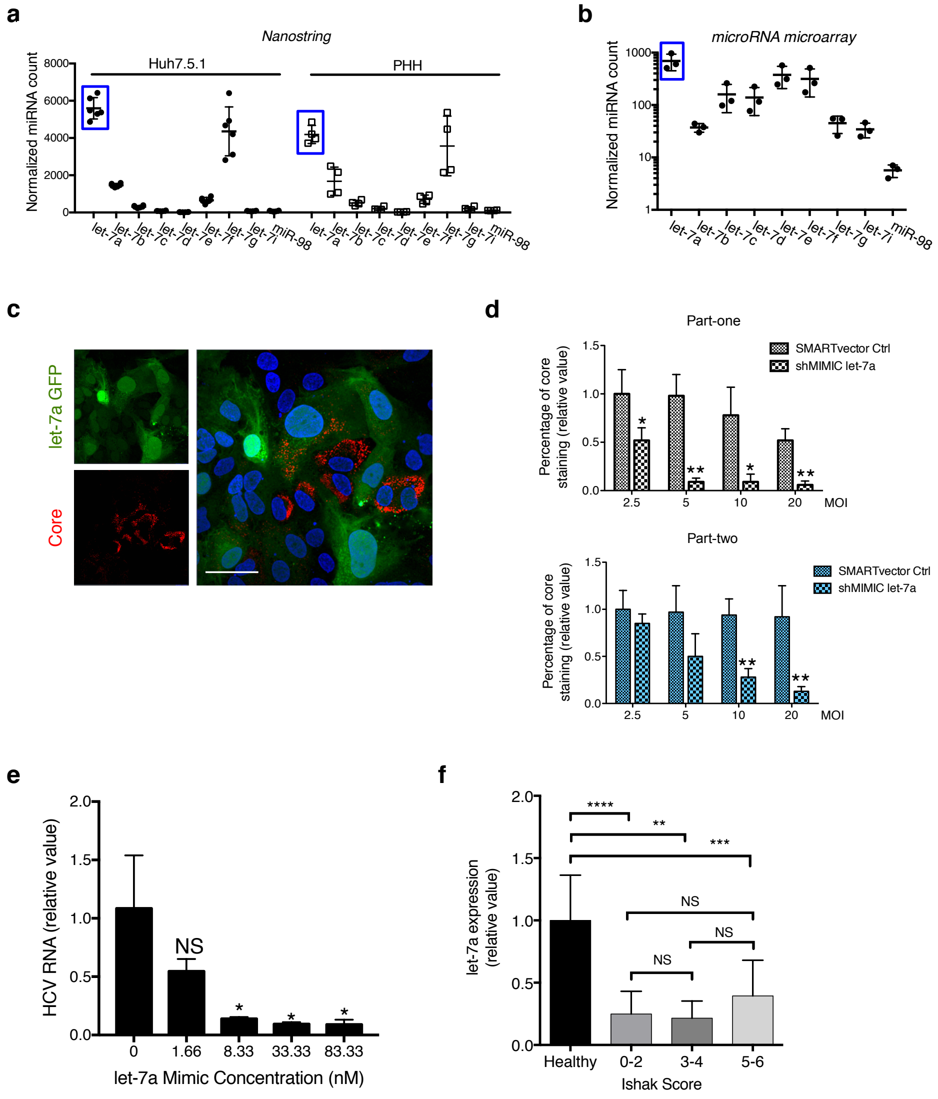

Supplementary Figure 8 let-7a restricts HCV infection in hepatocytes. (**a**,**b**) Expression levels of let-7 family members in Huh7.5.1 cells and primary human hepatocytes (PHH), quantified by NanoString nCounter assays (**a**); or in Huh7.5.1 cells, determined by miRNA microarray analysis (**b**). let-7a expression is highlighted in blue rectangle. Error bars represent mean ± s.d.
(**c**) shMIMIC lentiviral TurboGFP let-7a expression (in green) inhibits HCV core production in Huh7.5.1 cells, examined by immunofluorescence/confocal microscopy. Red, HCV core; blue, nuclei. Scale bars, 100 µm. (**d**) Quantitative analyses of part-one and part-two HCV core istaining in Huh7.5.1 cells infected with SMARTvector control or shMIMIC lentiviral let-7a at various indicated MOI. (**e**) Effects of let-7a synthetic mimic transfection at various indicated concentrations on levels of HCV RNA in Huh7.5.1 cells. (**f**) Hepatic let-7a expression is irrelevant to the fibrosis stages, assessed by Ishak score. (**d**–**f**) Values are normalized relative to SMARTvector control at the MOI of 2.5 (**d**), the mimic control (**e**), or healthy donor liver biopsies (**f**) as 1, and error bars represent SD of the mean, n = 3 (**d**,**e**), or 15 (healthy), 7 (Ishak Score 0-2), 3 (Ishak Score 0-2), or 8 (Ishak Score 0-2) (**f**). *P < 0.05, **P < 0.01 determined by Student's t test. NS, not significant.

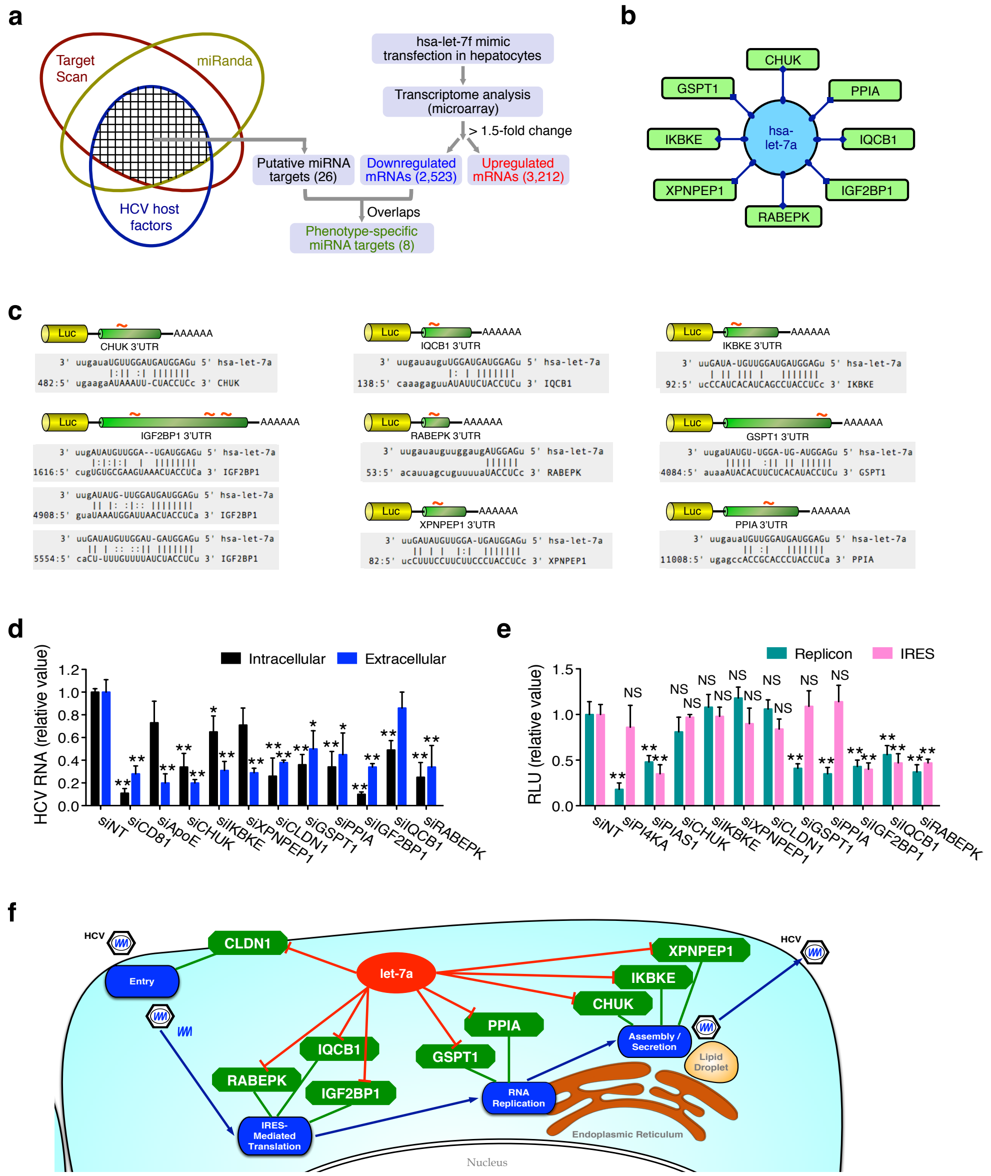

Supplementary Figure 9 Identification of let-7a cellular targets that mediate its functions in multiple stages of HCV infection. (**a**) Schematic of the strategy for systematic identification of putative let-7a phenotype-specific cellular targets. (**b**) List of HCV host factors identified as putative let-7a targets using above-described method. (**c**) Luciferase reporter-harboring 3’UTR constructs of various putative let-7a targets. Each 3’UTR encodes one or more let-7a seed matching sites. (**d**) Quantification of intracellular and extracellular HCV RNA in Huh7.5.1 cells treated with siRNAs against various indicated let-7a targets. (**e**) Depletion of various let-7a targets by siRNAs exerted effects on HCV replicon activity or IRES-mediated translation to various extents. (**f**) Schematic map illustrating that let-7a (in red oval) targets HCV host dependency factors (in green octagons) to exerts its function in restricting multiple stages of the HCV life cycle. Values are normalized relative to siNT, and error bars represent SD of the mean, n = 3 (**d**) or 5 (**e**). **P* < 0.05, ***P* < 0.01 determined by Student’s *t* test. NS, not significant.

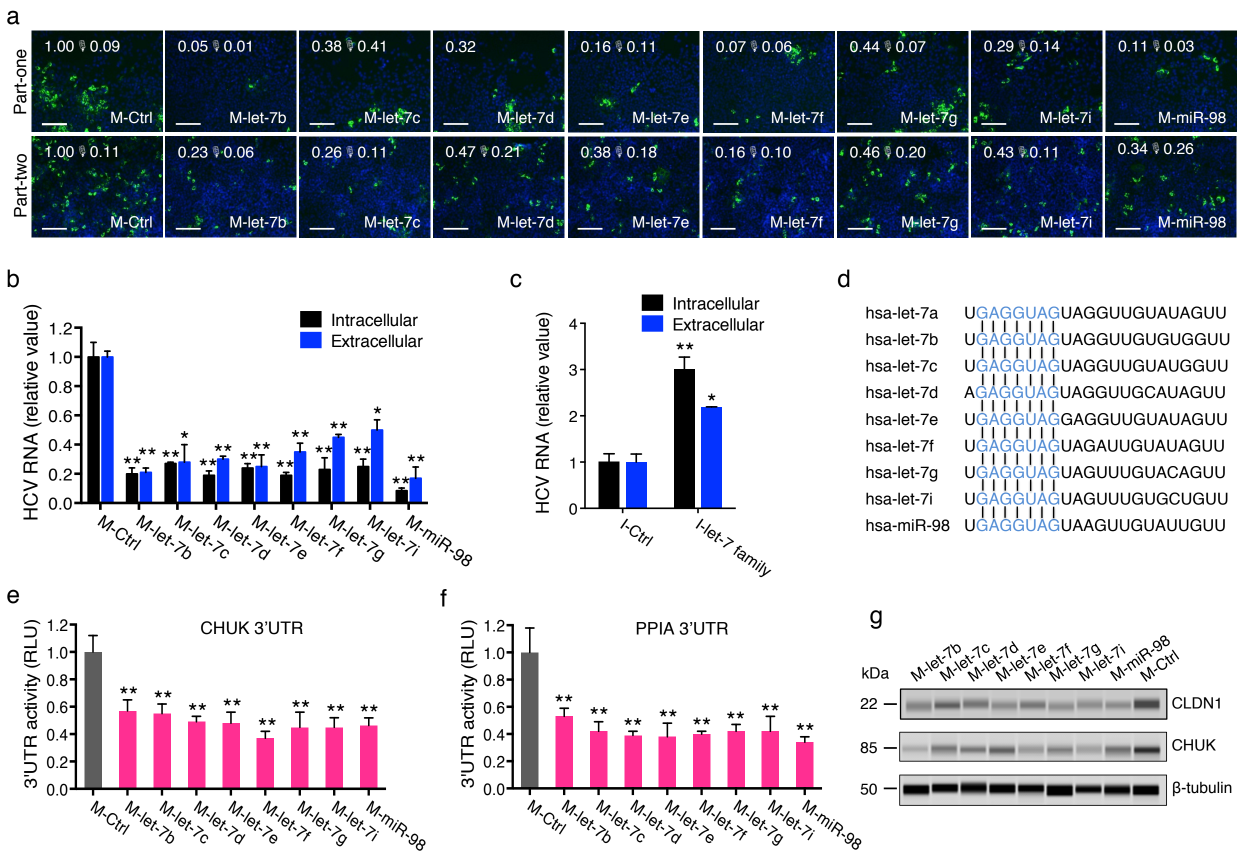

Supplementary Figure 10 let-7 family of miRNAs function similarly to let-7a in inhibiting HCV infection. (**a**,**b**) Effects of various let-7 family members on productive HCV infection in Huh7.5.1 cells, determined by HCV core staining (**a**) and quantification of intracellular and secreted viral RNA (**b**). (**a**) Green, HCV core; blue, nuclei. Scale bars, 100 μm. The numbers represent the percentages of core staining–positive cells. (**c**) Inhibition of all let-7 family members enhances HCV infection. Intracellular and extracellular HCV RNA levels were measured by Q-PCR. (**d**) Sequence alignments of let-7 family of miRNAs. Shared identical seed sequence is featured in blue. (**e**,**f**) Overexpressing the mimic of each let-7 family member suppresses 3'UTR activities of CHUK (**e**) and PPIA (**f**). (**g**) let-7 miRNA transfection reduces CLDN1 and CHUK protein levels. kDa: kilo Daltons. Values are normalized relative to the mimic or inhibitor control, and error bars represent SD of the mean, n = 3 (**b**,**c**) or 5 (**e**,**f**). **P* < 0.05, ***P* < 0.01 determined by Student's *t* test.

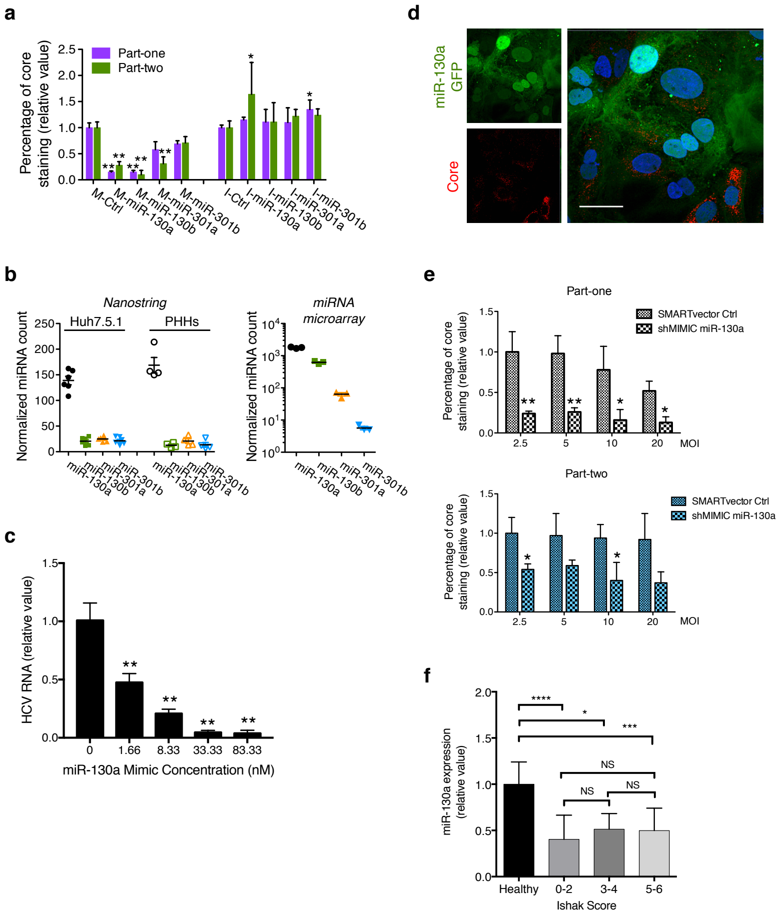

Supplementary Figure 11 Functions of miR-130 miRNAs in modulating HCV infection and their expression in hepatocytes. (**a**) Phenotypes of various miR-130 family members on HCV core production, measured in the primary miRNA mimic and inhibitor screens (see **Supplementary Tables 1** and **2**). Graphs depict percentages of cells stained positive for HCV core protein. Values are normalized relative to the mimic or inhibitor control, and represent mean ± s.d., n = 3. *P < 0.05, **P < 0.01 determined by Student's *t* test. (**b**) Expression patterns of miR-130 family members in Huh7.5.1 cells and PHH determined by NanoString technology, and in Huh7.5.1 cells determined by microarray analysis. Error bars represent ± SD of the mean, n=6 (Huh7.5.1 Nanostring), 4 (Nanostring PHHs), or 3 (microarray). (**c**) Transfection of miR-130a synthetic mimic at various indicated concentrations decreased HCV RNA levels in Huh7.5.1 cells. (**d**) shMIMIC lentiviral TurboGFP miR-130a expression (in green) restricted HCV infection in Huh7.5.1 cells, examined by immunofluorescence/confocal microscopy. Red, HCV core; blue, nuclei. Scale bars, 100 μm. (**e**) Quantitative analyses of part-one and part-two HCV core staining in Huh7.5.1 cells infected with SMARTvector control or shMIMIC lentiviral miR-130a at various MOI as indicated. (**f**) HCV-mediated downregulation of hepatic miR-130a expression is unrelated to liver fibrosis stages, indicated by Ishak score. (**c**,**e**,**f**) Values are normalized relative to the mimic control (**c**), SMARTvector control at the MOI of 2.5 (**e**) or healthy donor liver biopsies (**f**) as 1, and error bars represent SD of the mean, n = 3 (**c**,**e**), or 15 (healthy), 7 (Ishak Score 0-2), 3 (Ishak Score 0-2), or 8 (Ishak Score 0-2) (**f**). *P < 0.05, **P < 0.01 determined by Student's *t* test. NS, not significant.

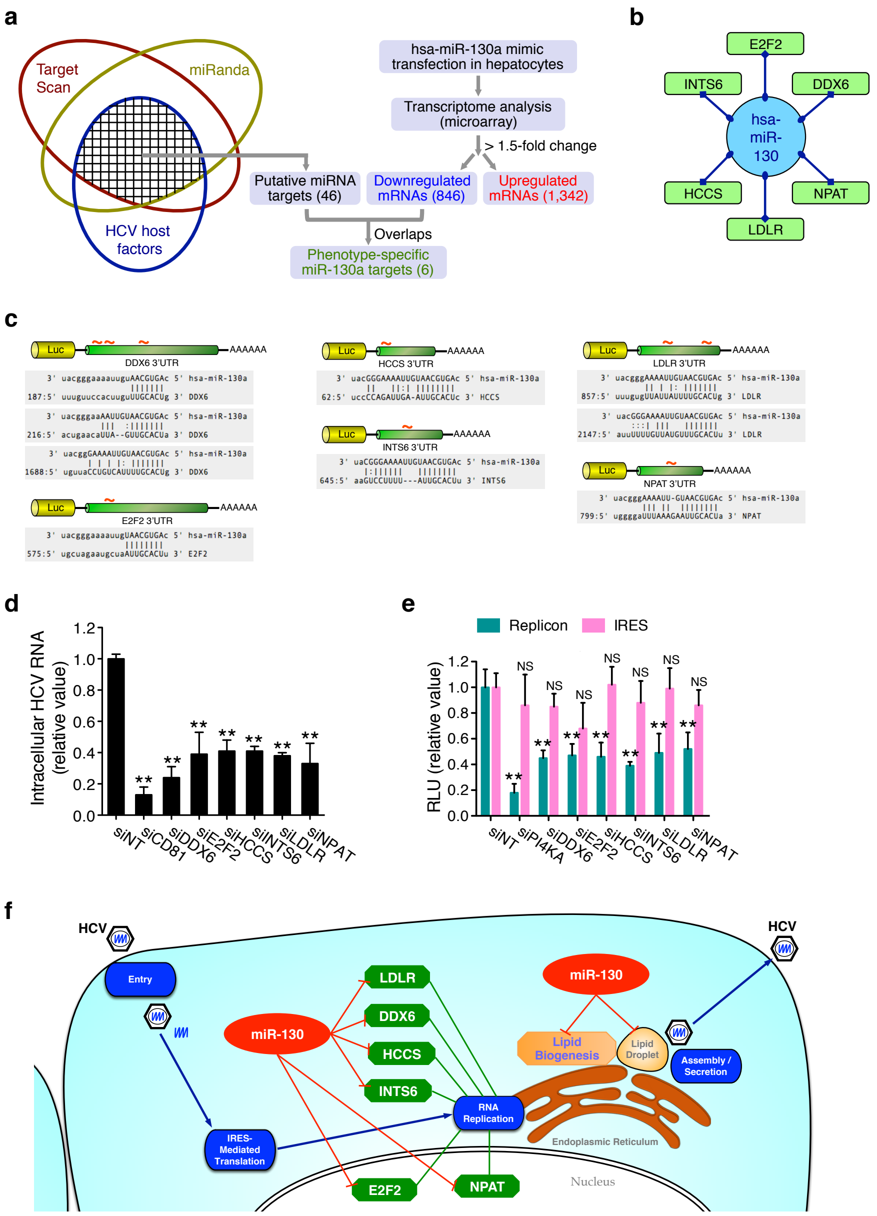

Supplementary Figure 12 Identification of miR-130 phenotype-related targets. (**a**) Schematics of the strategies for systematic identification of phenotype-specific miR-130 targets. (**b**) List of phenotype-specific, putative miR-130 targets, identified by the above-described method. (**c**) The 3'UTR of each putative miR-130a target possesses one or more miR-130a seed matching sites. (**d**,**e**) siRNA-mediated knockdown of various miR-130a targets diminishes HCV RNA production (**d**) and replicon activity (**e**) to various extents, but has no effect on HCV IRES-mediated translation (**e**). Error bars represent SD of the mean, n=3 (**d**) or 5 (**e**). **P < 0.01 determined by Student's t test. NS, not significant. (**f**) Schematic map demonstrating that miR-130 (in red oval) directly targets and represses the expression of multiple HCV proviral factors (in green octagons) for its inhibitory effect on HCV RNA replication. miR-130 also restricts HCV assembly by suppressing hepatocellular lipogenesis and LD formation.

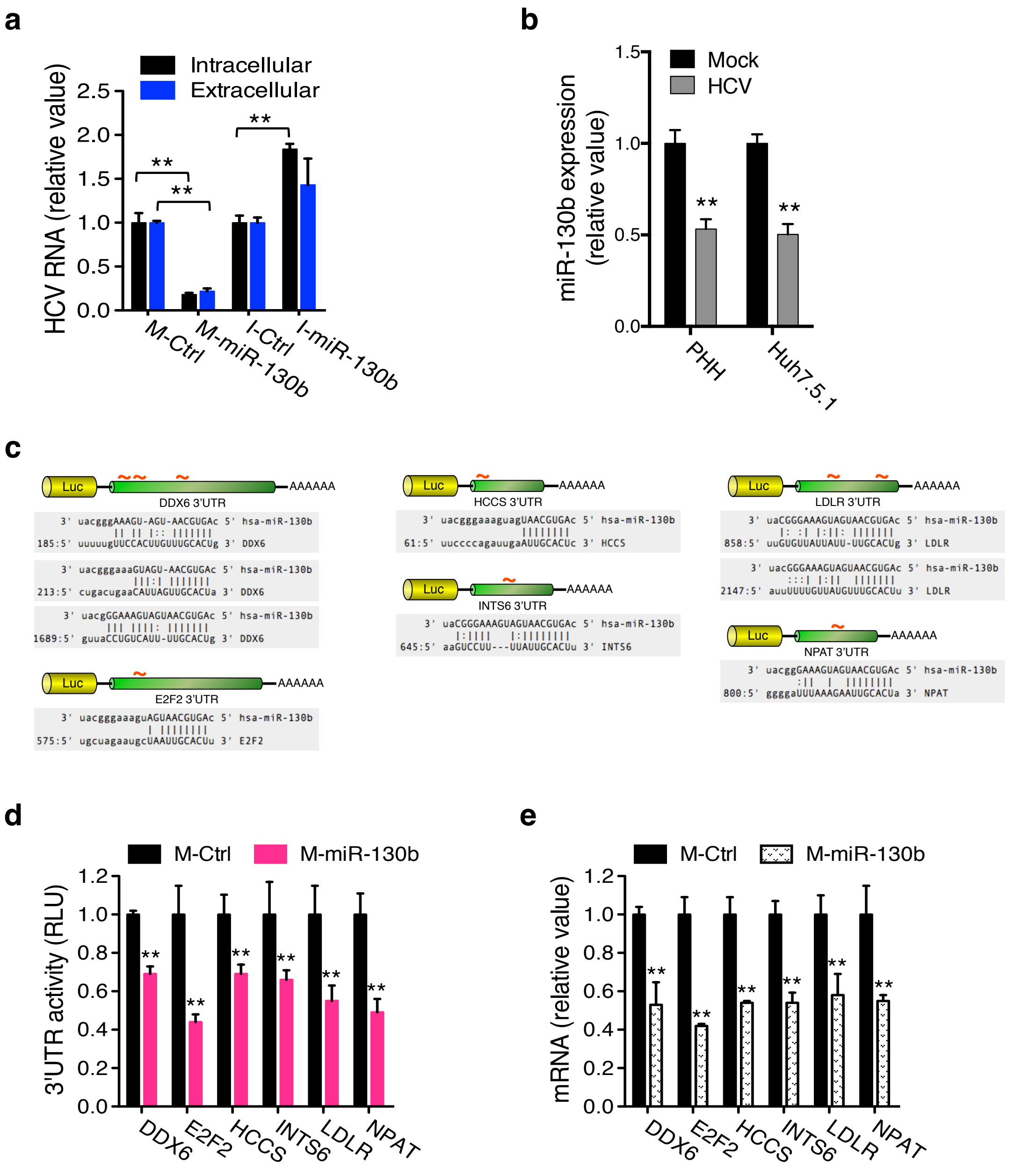

Supplementary Figure 13 Interaction of miR-130b with HCV infection and viral host dependencies. (**a**) Effects of miR-130b mimic or hairpin inhibitor on HCV RNA production in Huh7.5.1 cells. (**b**) HCV infection downregualtes miR-130b expression in Huh7.5.1 cells and PHH. (**c**) miR-130b putative targets possess one or more miR-130b seed matching sites on their 3'UTRs. (**d**,**e**) Overexpression of miR-130b mimic inhibits 3'UTR activities (**d**) and mRNA levels (**e**) of various cellular targets. Values are normalized relative to Ctrl, and error bars represent SD of the mean, n = 3 (**a**,**b**,**e**) or 5 (**d**). ***P* < 0.01 determined by Student's *t* test.

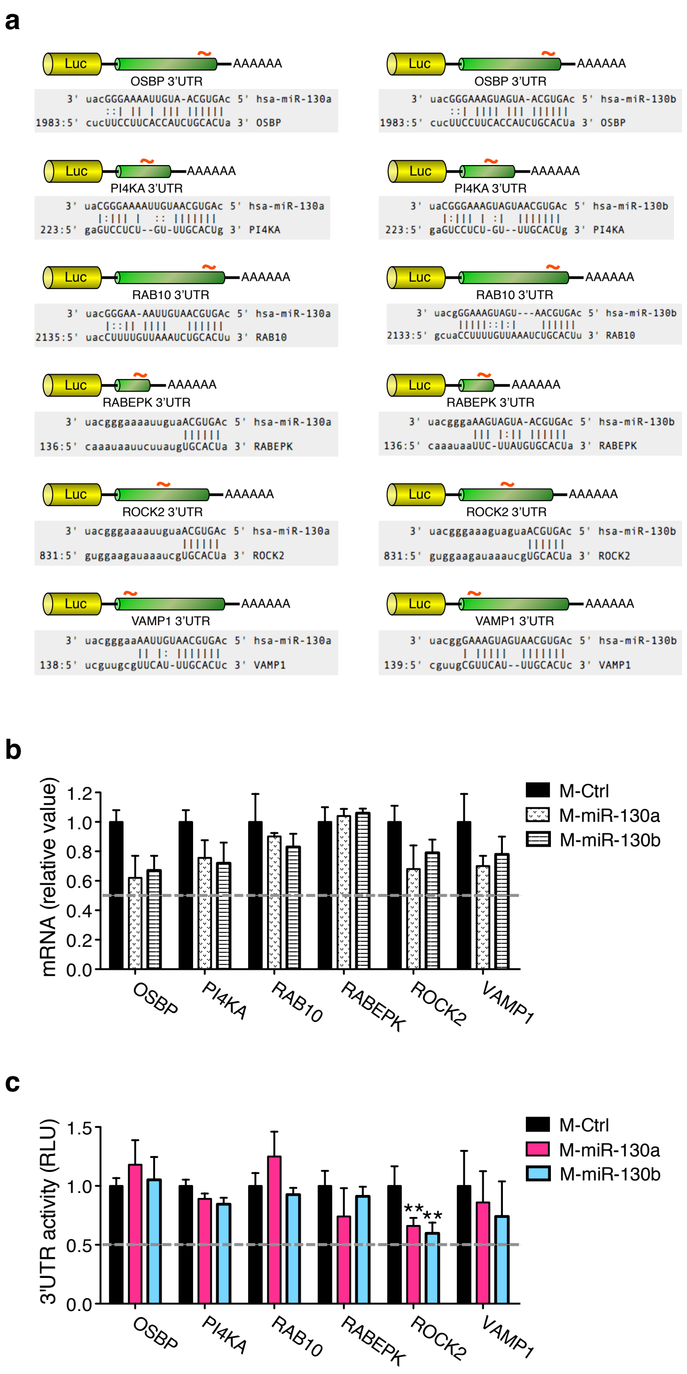

Supplementary Figure 14 Multiple HCV dependency factors with seed matching sites at their 3'UTRs are not direct miR-130 targets. (**a**) Illustration of miR-130a/b seed sequence matches within 3'UTRs of various HCV host factors. (**b**) miR-130a or miR-130b mimic transfection has no effect on mRNA levels of various putative bioinformatics-predicted targets. (**c**) miR-130a or miR-130b mimic overexpression does not or only moderately (for ROCK2) inhibits 3'UTR activities of the predicted targets. Values are normalized relative to the mimic Ctrl, and error bars represent SD of the mean, n = 3 (**b**) or 5 (**c**). $^{**}P < 0.01$ determined by Student's *t* test.

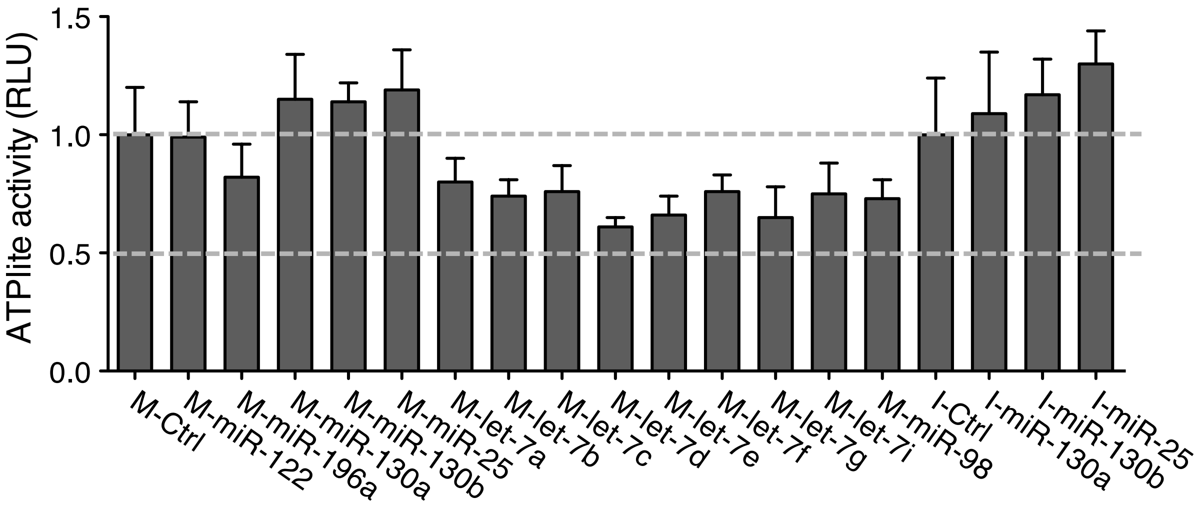

Supplementary Figure 15 Viability of cells treated with each indicated miRNA. Huh7.5.1 cells were treated with each indicated miRNA mimic or inhibitor that was studied in depth, ATPlite assays that examine potential cytotoxicity of the miRNAs were subsequently conducted. Values are normalized relative to the mimic or inhibitor Ctrl, and error bars represent SD of the mean, n = 5.

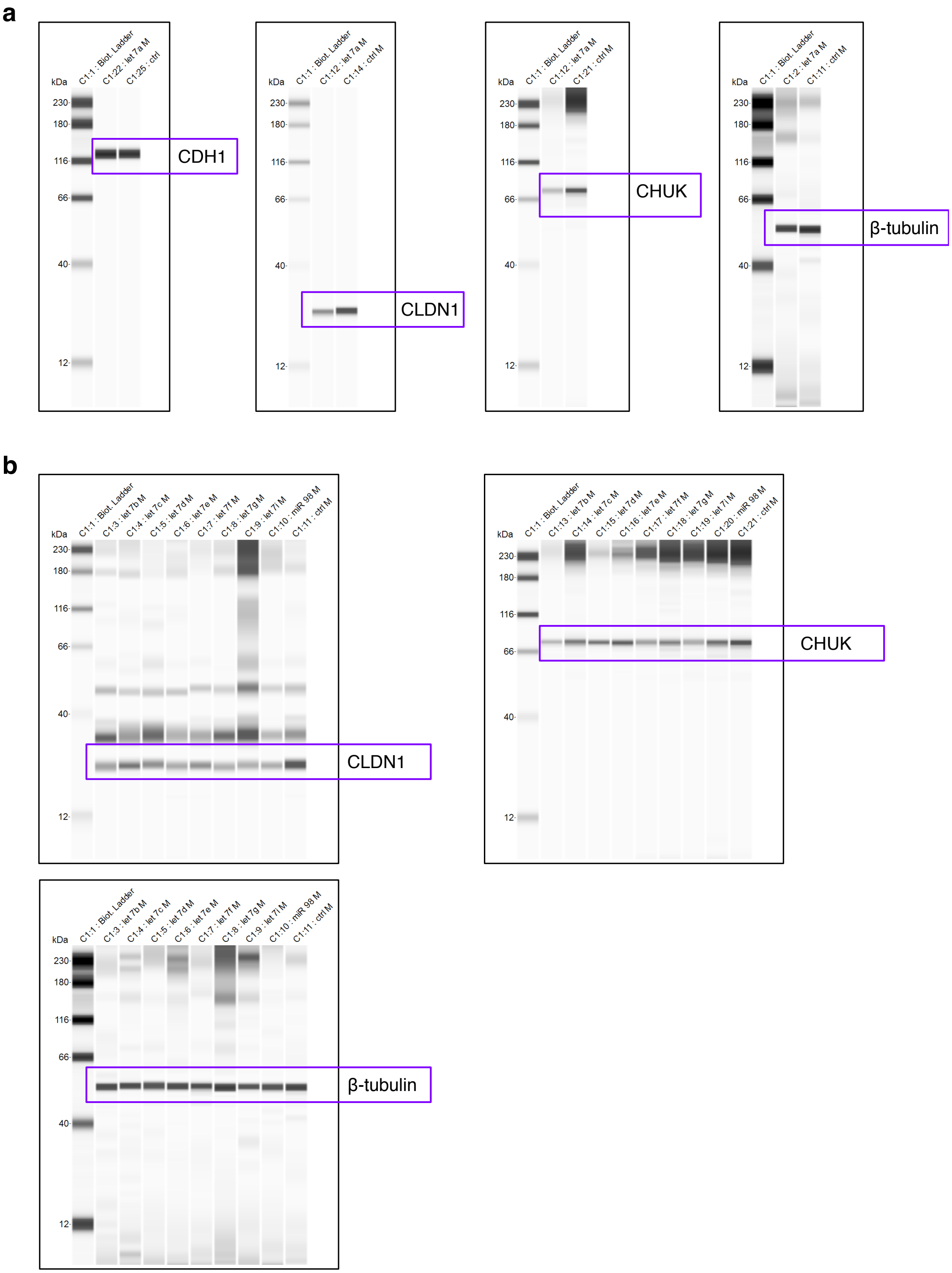

Supplementary Figure 16 Uncropped images of Western blots. (**a**) Uncropped Western blots found in **Fig. 5l**. (**b**) Uncropped Western blots found in Supplementary **Fig. 10g**. kDa: kilo Daltons.
