# Supplementary material for: Cellular microRNA networks regulate host dependency of hepatitis C virus infection
Source: Nat Commun. 2017 Nov 27;8:1789. doi: 10.1038/s41467-017-01954-x (PMC5702611; doi:10.1038/s41467-017-01954-x)
Supplement: Supplementary file 3 — Description of Additional Supplementary Files [file 41467_2017_1954_MOESM3_ESM.pdf]

File Name: Supplementary Data 1
Description: Genome-wide miRNA mimic screen to identify cellular miRNAs modulating productive HCV infection. HCV core immunostaining-based, miRNA gain-of-function assays were performed in Huh7.5.1 cells.

File Name: Supplementary Data 2
Description: Genome-wide miRNA hairpin inhibitor screen to confirm cellular miRNAs associated with HCV infection in hepatocytes. HCV core immunostaining-based, miRNA loss-of-function assays were performed.

File Name: Supplementary Data 3
Description: miRNA transcriptome analysis based on NanoString nCounter digital quantification of miRNA counts in Huh7.5.1 cells, in the absence or the presence of HCV infection.

File Name: Supplementary Data 4
Description: Description: miRNA transcriptome analysis based on NanoString nCounter digital quantification of miRNA counts in primary human hepatocytes (PHHs), in the absence or the presence of HCV infection.

File Name: Supplementary Data 5
Description: Genome-wide miRNA microarray analysis in Huh7.5.1 cells mock infected or infected with HCV for 48 h.

File Name: Supplementary Data 6
Description: Lists of HCV host dependencies that are also predicted targets of the 12 validated, HCV-relevant cellular miRNAs through bioinformatics analyses.

File Name: Supplementary Data 7
Description: Transcriptome analyses of Huh7.5.1 cells transfected with miR-25, let-7f, or miR-130a mimic.
